# Thermogravimetric and thermo-kinetic analysis of sugarcane bagasse pith: a comparative evaluation with other sugarcane residues

**DOI:** 10.1038/s41598-024-52500-x

**Published:** 2024-01-24

**Authors:** Hamidreza Najafi, Ahmad Golrokh Sani, Mohammad Amin Sobati

**Affiliations:** 1 XThermo Research Group, No.117, Somayeh Street, Tehran, 158176-8511 Iran; 2 School of Chemical Engineering, Iran University of Science and Technology (IUST), Postal Box 16765-163, Tehran, Iran

**Keywords:** Chemical engineering, Energy, Physical chemistry, Renewable energy

## Abstract

In this study, thermogravimetric and thermo-kinetic analysis of sugarcane bagasse pith (S.B.P.) were performed using a robust suite of experiments and kinetic analyses, along with a comparative evaluation on the thermo-kinetic characteristics of two other major sugarcane residues, namely sugarcane straw (S.C.S.) and sugarcane bagasse (S.C.B.). The thermogravimetric analysis evaluated the pyrolysis behavior of these residues at different heating rates in a nitrogen atmosphere. The Kissinger, advanced non-linear isoconversional (ANIC), and Friedman methods were employed to obtain effective activation energies. Moreover, the compensation effect theory (CE) and combined kinetic analysis (CKA) were used to determine the pre-exponential factor and pyrolysis kinetic model. Friedman's method findings indicated that the average activation energies of S.C.S., S.C.B., and S.B.P. are 188, 170, and 151 kJ/mol, respectively. The results of the ANIC method under the integral step Δα = 0.01 were closely aligned with those of the Friedman method. The CKA and CE techniques estimated *ln*(*f*(α)*A*_α_) with an average relative error below 0.7%. The pre-exponential factors of S.C.S., S.C.B., and S.B.P. were in the order of 10^14^, 10^12^, and 10^11^ (s^−1^), respectively. From a thermodynamic viewpoint, positive ∆G* and ∆H* results provide evidence for the non-spontaneous and endothermic nature of the pyrolysis process, indicating the occurrence of endergonic reactions.

## Introduction

Over the past few decades, the availability of agro-food wastes, particularly sugarcane by-products (S.C.B.) and waste (S.C.S. and S.B.P.), in large quantities on one hand, and the global tendency to address the energy crisis and invest in the renewable bioenergy sector, on the other hand, have led the scientists to identify a number of traditional and sophisticated thermochemical pathways to utilize and convert lignocellulosic materials, commonly referred to as biomass, into higher value-added products^1,2^. Among the various thermochemical processes, researchers have highly considered and evaluated the pyrolysis process^3–6^.

Pyrolysis of biomass and other degradable compounds is a multi-scale complicated process consisting of a large number of physicochemical interactions^7–9^. The physical changes at micro/particle and macro/reactor scales during pyrolysis can be described using the comprehensive transport phenomena formulations, while chemical conversions are predicted using various simple to detailed kinetic models^10–12^. Understanding the pyrolysis kinetics of a feedstock is important for the design, optimization, control, feasibility assessment, and scale-up of the pyrolysis reactors in industrial applications^13,14^.

In recent decades, several studies have used thermal analysis techniques such as differential scanning calorimetry (DSC) and thermogravimetric analysis (TGA) to collect non-isothermal data and identify reaction mechanisms and compute kinetic parameters of thermal conversion processes^15–20^.

Generally, two commonly employed approaches to mathematical explanation for solid-state decomposition kinetics are model-free and model-fitting techniques. Model-fitting methods are the most frequently used to describe the conversion rate of the pyrolysis feed to product (i.e., global model) or a set of pseudo-component products (i.e., semi-global model). Pre-assumed reaction models and collected experimental data are used to approximate distinct kinetic parameters (also known as ‘kinetic triplets’) for each reaction through the application of linear or non-linear regression techniques^21–23^. Usually, the conversion mechanism and its reaction model differ depending on the feedstock properties and the reaction conditions.

In contrast to the model-fitting approach, model-free methods, commonly referred to as isoconversional methods^24,25^, operate under the premise that, at a predetermined conversion level, temperature is the sole variable influencing the reaction rate^26^. This implies that the activation energy (Ea) values for each specific conversion can be directly obtained without making assumptions about the underlying nature of the reaction model^27^.

Within the well-known realm of "model-free" methodologies, the determination of activation energy involves employing either a linear or nonlinear isoconversional procedure. The choice between these approaches depends on the assumptions underlying the selection of integral or differential isoconversional methods. It is worth mentioning that the differential isoconversional methods, owing to their reliance on instantaneous rate values, are susceptible to experimental noise, resulting in numerical instability. This issue can be effectively mitigated by adopting integral isoconversional approaches^22,28^.

Moreover, the pre-exponential factor (*A*) can be determined with noteworthy precision using sophisticated methods that adhere to a model-free approach^29–32^. Subsequently, the identification of activation energy and the pre-exponential factor allows for the generation of a tabular representation depicting an explicit version of the reaction model^27,32^.

International Confederation for Thermal Analysis and Calorimetry (ICTAC) kinetic researches shows that both model-fitting and isoconversional approaches can adequately describe the kinetics of single-step and multi-step processes, provided that the models in the model-fitting method are simultaneously fitted to several datasets gathered under various temperature programs^21,22,33^. However, when modeling multi-step reactions using model-fitting methods, various nontrivial issues arise, which is not the case when employing the model-free approaches^22^. In other words, isoconversional techniques can reduce the risk of mistakes in the model selection and parameter estimation.

Estimating the kinetic parameters of the pyrolysis process often involves the utilization of a model-free approach, which encompasses several methods, including Kissinger^34^, as well as various differential and integral isoconversional techniques such as Friedman^35^, Ozawa^36^, Ozawa-Flynn-Wall (OFW)^36,37^, Kissinger–Akahira–Sunose (KAS)^38^, and advanced non-linear (NLN) isoconversional (ANIC) methods^39,40^.

It was shown by Vyazovkin et al.^27,28^ that the OFW and KAS methods might produce relatively good values of the activation energy at *E/RT* > 13 due to the approximations made in these approaches. They also demonstrated that the ANIC and Friedman approaches are essentially independent of the *E/RT* value and can produce exceptionally low errors in the activation energy. As a result, ANIC and Friedman methods can be recommended to compute the activation energy necessary for the predictions and solve the other issues sensitive to activation energy accuracy^27,28^.

The pyrolysis kinetics of various biomass and agriculture waste materials have been extensively studied in the literature. These materials include sugarcane straw^41^, sugarcane bagasse^41^, soybean hull^17^, rice and corn^18^, tobacco waste^42^, plum and fig pomace^43^, olive mill solid waste^44^, coconut shell straws^45^, poplar wood^46^, pinewood sawdust^47^, bamboo sawdust^48^, maize straw, invasive lignocellulosic biomasses (i.e., Prosopis juliflora and Lantana camara)^49^ and digested organic fractions^50^. These studies employ a wide range of techniques, both model-fitting and model-free methods such as Kissinger, KAS, OFW, Friedman, ANIC, distributed activation energy model (DAEM) and other hybrid approaches^51^. To the best of our knowledge, the kinetic analysis of the pyrolysis reaction for S.B.P. has not been specifically discussed in the literature.

In this study, thermogravimetric and thermo-kinetic analysis of sugarcane bagasse pith (S.B.P.) was performed using a robust suite of experiments and kinetic analyses along with a comparative evaluation on the thermo-kinetic characteristics of two other major sugarcane residues, namely sugarcane straw (S.C.S.) and sugarcane bagasse (S.C.B.). In this regard, the thermogravimetric analysis is used to evaluate the pyrolysis behavior of sugarcane residues at seven distinct heating rates in a nitrogen atmosphere. The Kissinger^34^, Friedman^35^, and ANIC^39,40^ methods were utilized to obtain the activation energies. Moreover, the compensation effect theory^29,32^ and the combined kinetic analysis^52^ were employed to determine the samples pre-exponential factor and pyrolysis kinetic model using TG data. Simultaneously, a procedural and repeatable workflow for analyzing the results of the selected convergent approaches, in terms of determining the proper pyrolysis reaction model and estimating the kinetic parameters, is proposed based on the successful agreement between the study outcomes and the experimental data.

## Materials and methods

### Materials and experimental methods

The materials and experimental procedures utilized in this study have been previously described in our recent publication^1^. Sugarcane residues were collected from the CP69-1062 variety obtained from Karun Agro-Industry in Iran's Khuzestan province. It is important to mention that these materials are waste or by-products of the harvesting, juice extraction, and milling industrial processes of sugarcane, and do not involve the collection or utilization of live sugarcane plants. The use of these industrial residues complies with relevant institutional, national, and international guidelines and legislation governing the utilization of agricultural waste products for research purposes. All samples were prepared according to ASTM E1757-01 (2015) standards. Dry samples were crushed using a Retsch PM 100 planetary ball mill, sieved to a particle size of ≤ 212 μm (US Mesh 70), and then analyzed using a Mettler-Toledo TGA 1 thermal analyzer under high-purity nitrogen at a flow rate of 50 mL/min. The heating protocol involved ramping the temperature at 10 °C/min up to 105 °C, followed by a 10 min hold and further heating to 800 °C. Heating rates ranging from 10 to 40 °C/min were tested with approximately 10.7 mg of each sample. The entire TGA procedure was repeated three times, with an average deviation of less than 1.45%.

### Theoretical methods

In thermogravimetric analysis (TGA), the degree of conversion (*α*) is defined as the mass fraction of the decomposed solid throughout the process. The degree of conversion (*α*) is calculated using the initial mass (*m*_0_), final residual mass (*m*_*f*_), and mass at any given time (*m*_*t*_) according to Eq. (1):

1 $$\alpha =\frac{{m}_{0}-{m}_{t}}{{m}_{0}-{m}_{f}}$$

Assuming that all of the components in solid or many condensed phases have the same reactivity and ignoring the influence of pressure on thermal analysis kinetics^22,53^, the kinetics of a single-step reaction can typically be described by the following rate equation. This equation can be considered as a product of two independent functions^22^:

2 $$\frac{d\alpha}{dt}=k\left(T\right)\cdot f\left(\alpha\right)$$

where in Eq. (2) *α* is the degree of conversion, *t* is the conversion time, *dα/dt* is the rate of the reaction process, *k*(*T*) is the reaction rate constant, *T* is the reaction temperature, and *f*(*α*) is a conversion function that demonstrates the reaction model used and relies on the controlling mechanism.

The effect of temperature on the reaction rate is typically assumed to follow the Arrhenius equation, as shown below^22^:

3 $$k\left(T\right)=A\mathit{exp}\left(\frac{-{E}_{a}}{RT}\right)$$

where *A*, *E*_*a*_ and *R* are the Arrhenius pre-exponential factor, the activation energy, and the universal gas constant, respectively.

In certain conditions, the non-isothermal reaction rate expressions can be represented under constant heating rate (*β*), alongside corresponding superficial transformation equations, as follows:

4 $$T={T}_{0}+\beta t$$

5 $$\frac{d\alpha}{dT}=\frac{d\alpha}{dt}\cdot \frac{dt}{dT}=\frac{1}{\beta}\frac{d\alpha}{dt}$$

By combining Eqs. (2), (3), and (5), Eq. (2) turns into:

6 $$\frac{d\alpha }{dt}=\beta \frac{d\alpha }{dT}=A\mathit{exp}\left(\frac{-{E}_{a}}{RT}\right)f\left(\alpha \right)$$

In the case where *A* remains a constant, the integral form of Eq. (6) can be represented by the following equation^22,28^:

7 $$g\left(\alpha \right)={\int }_{0}^{\alpha }\frac{d\alpha }{f\left(\alpha \right)}=A{\int }_{0}^{t}\mathit{exp}\left(\frac{-{E}_{a}}{RT}\right)dt=\frac{A}{\beta }{\int }_{{T}_{0}}^{T}\mathit{exp}\left(\frac{-{E}_{a}}{RT}\right)dT$$

Alternatively, Eq. (7) can be concisely reformulated as the temperature integral function:

8 $$g\left(\alpha \right)=A\left(J\left({E}_{a},T\left(t\right)\right)\right)=\frac{A}{\beta }\left(I\left({E}_{a},T\right)-I\left({E}_{a},{T}_{0}\right)\right)$$

In these expressions, *g*(*α*) signifies the integral representation of the reaction model, while *I*(*E*_*a*_*, T*) or* J*(*E*_*a*_*, T*(*t*)) denote temperature integral functions*.* Table 1 comprises a well-known set of the reaction models and their integral counterparts, highlighting the dependence on *α* in the reaction kinetics^22,32^. Equations (6) and (7) are considered as the basic equations of differential and integral methods, respectively.

**Table 1:** Common reaction models for the reaction kinetics in solid-state reactions.

No	Reaction model	Symbol	*f* (*α*)	*g* (*α*)
1	Mampel (first order)	A1, F1	1 − α	− ln(1 − α)
2	Chemical reaction (second order)	F2	(1 − α)^2^	(1 − α)^−1^–1
3	Chemical reaction (third order)	F3	(1 − α)^3^	[(1 − α)^−2^–1]/2
4	Avrami–Erofeev	A2	2(1 − α) [− ln (1 − α)]^1/2^	[− ln (1 − α)]^1/2^
5	Avrami–Erofeev	A3	3(1 − α) [− ln (1 − α)]^2/3^	[− ln (1 − α)]^1/3^
6	Avrami–Erofeev	A4	4(1 − α) [− ln (1 − α)]^3/4^	[− ln (1 − α)]^1/4^
7	One-dimensional diffusion	D1	1/2 α^−1^	α^2^
8	Two-dimensional diffusion	D2	[− ln (1 − α)]^−1^	(1 − α) ln (1 − α) + α
9	Three-dimensional diffusion	D3	3/2 (1 − α)^2/3^[1 − (1 − α)^1/3^]^−1^	[1 − (1 − α)^1/3^]^2^
10	Power law	P2	2α^1/2^	α^1/2^
11	Power law	P2/3	2/3 α^−1/2^	α^3/2^
12	Power law	P3	3α^2/3^	α^1/3^
13	Power law	P4	4α^3/4^	α^1/4^
14	Contracting cylinder	R2	2(1 − α)^1/2^	1 − (1 − α)^1/2^
15	Contracting sphere	R3	3(1 − α)^2/3^	1 − (1 − α)^1/3^

If the degree of conversion is kept constant, then *f* (*α*) is fixed at any temperature or temperature regime. In this scenario, the process mechanism becomes exclusively dependent on the conversion instead of the temperature^22,28^. Isoconversional methods, based on these assumptions, facilitate the estimation of activation energy without being constrained by a specific reaction model.

In accordance with the selected approach and associated hypotheses, various isoconversional methods have been extensively detailed in existing literature^32,34,35,37–40^. These techniques commonly determine the activation energy at a predetermined conversion degree using data obtained through a series of TG runs.

#### Kissinger method

The Kissinger method relies on obtaining the maximum reaction peak temperature and the corresponding maximum reaction rate from each heating rate series generated by thermal analysis instruments, such as DSC and TGA. The method's basic equation is Eq. (6), where the maximum rate takes place when *d*^2^*α/dt*^2^ is zero^22,34^:

9 $$\frac{{d}^{2}\alpha }{d{t}^{2}}=\left[\frac{{E}_{a}\beta }{R{T}_{m}^{2}}+A{f}{\prime}\left({\alpha }_{m}\right)\mathit{exp}\left(\frac{-{E}_{a}}{R{T}_{m}}\right)\right]{\left(\frac{d\alpha }{dt}\right)}_{m}=0$$

here, *f*′(*α*) = *df*(*α*)*/dt,* and the subscript *m* specifies the variables related to the maximum reaction rate. If the reaction model is assumed to be first order (Table 1) and the natural logarithm is used, Eq. (9) can be stated as follows^22,34^:

10 $$ln\left(\frac{\beta }{{T}_{m,i}^{2}}\right)=ln\left(\frac{AR}{{E}_{a}}\right)-\frac{{E}_{a}}{R{T}_{m,i}}$$

where *i* represents the index of the individual heating rate (*β*). The activation energy (*E*_*a*_) and pre-exponential factor (*A*) can be determined by plotting *ln*(*β/T*^2^_*m,i*_) against *1/T*_*m,i*_ and fitting all the data with a straight line.

#### Friedman method

Performing a natural logarithmic transformation to Eq. (6) gives:

11 $${\mathit{ln}\left(\frac{d\alpha }{dt}\right)}_{\alpha ,i}=\mathit{ln}\left[f\left(\alpha \right){A}_{\alpha }\right]-\frac{{E}_{a}}{R{T}_{\alpha ,i}}$$

in Eq. (11), known as Friedman’s method^35^, *i* is the individual heating rate (*β*) index, and *T*_*α,i*_ is the temperature at which the degree of conversion *α* is accomplished. For any specified value of *α*, the slope of a plot of *ln*(*dα/dt*)_*α,i*_ against *1/T*_*α,i*_ yields the value of *E*_*a*_, and the mathematical function *f*(*α*), which describes the reaction model, can be determined from the intercept of the plot. Equation (11) can be used with any temperature program, in addition to its applicability to linear heating programs.

#### Advanced NLN isoconverstional method

Integrating Eq. (7) over small time intervals (*t*_α-Δα_) → *t*_α_ provides the following results for each given value of α^27,40^:

12 $$g{\left(\alpha \right)}_{\alpha }=A{\int }_{{t}_{\alpha -\Delta \alpha }}^{{t}_{\alpha }}\mathit{exp}\left(\frac{-{E}_{a,\alpha }}{RT\left(t\right)}\right)dt=\frac{A}{\beta }{\int }_{{T}_{\alpha -\Delta \alpha }}^{{T}_{\alpha }}\mathit{exp}\left(\frac{-{E}_{a,\alpha }}{RT}\right)dT$$

Equation (12) lacks a fully analytical solution; hence *E*_*a,α*_ must be calculated numerically. Following the isoconversional method assumptions, it is presumed that *g*(*α*) is constant at equivalent conversions (at each heating rate). In other words, the response reaction model remains mostly unchanged and maintains a consistent structure at the given conversion. Consequently, based on Eqs. (8) and (12), for a particular conversion and relying on the outcomes of a series of experiments conducted at a discrete heating rate (*β*_i_), the following can be expressed:

13 $$J\left({E}_{a,\alpha },T\left({t}_{\alpha }\right)\right)={\int }_{{t}_{\alpha -\Delta \alpha }}^{{t}_{\alpha }}\mathit{exp}\left(\frac{-{E}_{a,\alpha }}{RT\left(t\right)}\right)dt$$

under a constant heating rate, one could express:

14 $$\frac{I\left({E}_{a,\alpha },{T}_{\alpha ,i}\right)}{{\beta }_{i}}={\int }_{{T}_{\alpha -\Delta \alpha }}^{{T}_{\alpha }}\mathit{exp}\left(\frac{-{E}_{a,\alpha }}{RT}\right)dT$$

This implies that for a given conversion and a set of experiments performed at *n*th arbitrary heating rates, the following results could be obtained, according to Eqs. (8) and (12):^27,54^

15 $$g{\left(\alpha \right)}_{\alpha ,{\beta }_{1}}=g{\left(\alpha \right)}_{\alpha {,\beta }_{2}}=\cdots =g{\left(\alpha \right)}_{\alpha {,\beta }_{n}}= \text{ constant}$$

or

16 $${A}_{\alpha }J\left({E}_{a,\alpha },{T}_{1}\left({t}_{\alpha }\right)\right)={A}_{\alpha }J\left({E}_{a,\alpha },{T}_{2}\left({t}_{\alpha }\right)\right)=\cdots ={A}_{\alpha }J\left({E}_{a,\alpha },{T}_{n}\left({t}_{\alpha }\right)\right)= \text{ constant}$$

Equation (16) can be generalized by dividing both sides of Eq. (15) by one another and summing. The result is^54^:

17 $$min\left|{\sum }_{i=1}^{n}{\sum }_{\begin{array}{c}j=1\\ j\ne i\end{array}}^{n}\frac{J\left({E}_{a,\alpha },{T}_{i}\left({t}_{\alpha }\right)\right)}{J\left({E}_{a,\alpha },{T}_{j}\left({t}_{\alpha }\right)\right)}\right|={\Omega }_{\alpha }=n\left(n-1\right)$$

In Eq. (17), the optimization indicator is designated by the symbol *Ω*_*α*_, signifying the minimum achievable value of the equation. Additionally, the temperature integral functions (*J*) can be calculated using the trapezoidal rule^40^, or the Senum and Yang approximation^27,39,55,56^. *E*_*α*_ can be determined as the value that minimizes *Ω*_*α*_ by repeating the optimization procedure for each *α* value using Eq. (17)^40,54^. Equation (17) is applicable to a wide range of temperature programs, and its accuracy depends on the size of the integral step^28,57^.

In the following, the integral methods can be employed to obtain the mathematical function that describes the reaction model through the application of Eq. (18):^31^

18 $${G\left(\alpha \right)=g\left(\alpha \right)-g\left(\alpha -\Delta \alpha \right)={\int }_{\alpha -\Delta \alpha }^{\alpha }\frac{d\alpha }{f(\alpha )}=}A_{\alpha }J\left({E}_{a,\alpha },T\left({t}_{\alpha }\right)\right)$$

By applying the presumption that the reaction model is constant over a small conversion interval and is independent of the heating rate to the integral function on the left side of Eq. (18), the mathematical function *f(α)* describing the reaction model can be obtained from Eq. (19):

19 $${A}_{\alpha }f\left(\alpha \right)=\frac{\Delta \alpha }{J\left({E}_{a,\alpha },T\left({t}_{\alpha }\right)\right)}$$

#### Determining the pre-exponential factor

Finding the reaction model and pre-exponential factor may be accomplished by combining the outcomes of a model-free approach and a model-fitting method for a particular heating rate^22^. Several methods exist in the model-free and isoconversional computations to estimate the pre-exponential factor. These methods can be classified as model-based or model-free^22,29^. Researchers^28–31^ have demonstrated that when the *E*_*α*_ varies significantly with *α*, as it does in a multi-step process, the application of model-free based procedures leads to outstanding results, notably for the Friedman and ANIC methods. The objective is to take advantage of the compensation effect (CE), expressed as follows^58^:

20 $$ln{A}_{i}=a{E}_{i}+b$$

In order to compute the pre-exponential factor, after evaluating *E*_*a*_ using an isoconversional technique, several values of *E*_*a*_ and *A* could be determined by the model-fitting method based on TG experimental data gathered at a single heating rate for each reaction model presented in Table 1. Finally, by utilizing these values and Eq. (20), and obtaining values for *a* and *b*, the pre-exponential factor can be calculated based on each pre-evaluated value of *E*_*a*_.

#### Identification of the kinetic model

Adaptable theoretical models are commonly utilized to accurately represent and justify deviations from idealized processes^28,59,60^. The most well-known model is a modified form of Sestak and Berggren's truncated equation (tSB)^22,52^.

Pérez-Maqueda^52^ revealed that the findings of the combined kinetic analysis (CKA) (i.e., *E*_*a*_, *A*, and *f*(*α*)), obtained from linear model-fitting of TG analysis data collected from arbitrary temperature programs to the tSB reaction model, could be reconciled with a number of theoretical reaction models (e.g., Table 1). This method has the advantage that the reaction model is not confined to Table 1 or comparable kinetic models. Considering the CKA approach, the following generic form is used to determine the kinetic model^52^:

21 $$f\left(\alpha \right)=c {\alpha }^{m}{\left(1-\alpha \right)}^{n}$$

In fact, Eq. (21) could be adjusted by modifying the values *c*, *n,* and *m* to match different ideal kinetic models developed under particular mechanistic assumptions^52^.

The combined kinetic analysis is relied on rearranging Eq. (6) and replacing *f*(*α*) with Eq. (21):

22 $$ln\left[\frac{d\alpha }{dt}\frac{1}{{\alpha }^{m}{\left(1-\alpha \right)}^{n}}\right]=\mathit{ln}\left(cA\right)-\frac{{E}_{a}}{RT}$$

The unknown kinetic parameters of Eq. (22) can be effectively determined using the nonlinear optimization method and thermogravimetric data collected at one or more heating rates. Afterwards, the optimization results are employed to evaluate the maximum correlation coefficient (R^2^). This assessment is carried out using the linear representation of Eq. (22), within specific conversion (*α*) ranges. The indicated evaluation provides the values of *n* and *m*, along with the intercept (*ln*(*cA*)) and slope (*E*_*a*_) of Eq. (22) over the specified conversion (*α*) range. If the values of *n* and *m* do not fall within the expected range of one of the ideal kinetic models (see Table 1), it becomes impossible to separate the variables *c* and *A* in the intercept (*ln*(*cA*)) of Eq. (22). However, studies have demonstrated that the impact of *c* on the pre-exponential factor, commonly denoted as *ln*(*A*), is negligible and can be disregarded under certain conditions due to the relatively small value of *c*^22^.

In conclusion, it can be stated that although the effect of conversion on the reaction rate as determined by the CKA method (i.e., *f*(*α*)) may not be entirely consistent with any ideal kinetic model, the results obtained can still be used to compare isoconversional methods for identifying a correct reaction model^22^.

#### Thermodynamic parameters calculation

The following general equation can be written using Eyring's active complex theory to derive the thermodynamic parameters^61^:

23 $$k\left(T\right)=\kappa \frac{{\kappa }_{B}T}{h}\mathit{exp}\left(\frac{\Delta {S}^{*}}{R}\right)\mathit{exp}\left(\frac{-\Delta {H}^{*}}{RT}\right)$$

In Eq. (23), $\frac{{\kappa }_{B}T}{h}$ is the frequency of vibration of the high-energy, active complex surmounting the transition state energy barrier^62^. The probability that a chemical reaction will occur once the system has attained the active state is indicated by the transmission coefficient (*κ*). The transmission coefficient quantifies the probability that a complex would dissociate into products rather than reactants^61,63^. Theoretically, *κ* might range from zero to one, but it often takes unity^61^.

Whether the reaction is monomolecular or bimolecular, the difference between ∆H* in Eq. (23) and *E*_*a*_ in Eq. (3) is only one or two *R.T.* values. Since this discrepancy typically falls within the expected range of experimental activation energy uncertainty, which is around 5–10%, it can be overlooked^29,64^. By comparing Eqs. (3) and (23) and assuming ∆H^*^≈ *E*_*a*_, the change of the activation entropy at the formation of an activated complex from the reactant is obtained:

24 $$\Delta {S}^{*}=R\mathit{ln}\left(\frac{Ah}{{\kappa }_{B}T}\right)$$

and,

25 $$\Delta {H}^{*}={E}_{a}-RT$$

The following thermodynamic relationship is used to calculate the Gibbs free energy of the activated complex formation:

26 $$\Delta {G}^{*}=\Delta {H}^{*}-T\Delta {S}^{*}$$

In contrast, by substituting Eq. (3) in Eq. (23) and considering Eq. (26), the value of $\Delta {G}^{*}$ can also be obtained from the following equation:

27 $$\Delta {G}^{*}={E}_{a}+\mathit{RT} \mathit{ln}\left(\frac{{\kappa }_{B}T}{Ah}\right)$$

According to the equations, two approaches for determining the thermodynamic parameters are compared in Eqs. (24)–(27). Method I, consists of Eqs. (24)–(26), while Method II consists of Eqs. (25)–(27). In Eqs. (23)–(27) *κ*_*B*_ is the Boltzmann constant (1.38065 × 10^–23^ J/K), *h* is the Planck constant (6.62607 × 10^–34^ J.s), the value of *κ* is assumed to be one, ∆S^*^ (J/mol.K), ∆H^*^ (kJ/mol) and ∆G^*^ (K.J./mol) are the entropy, enthalpy and Gibbs free energy of activation, respectively. All thermodynamic parameters based on Method I or II can be obtained by substituting *T* = *T*_*p*_, the maximum decomposition temperature from the differential thermogravimetry (DTG) data, in Eqs. (24)–(27).

## Results and discussion

**Figure 1:**
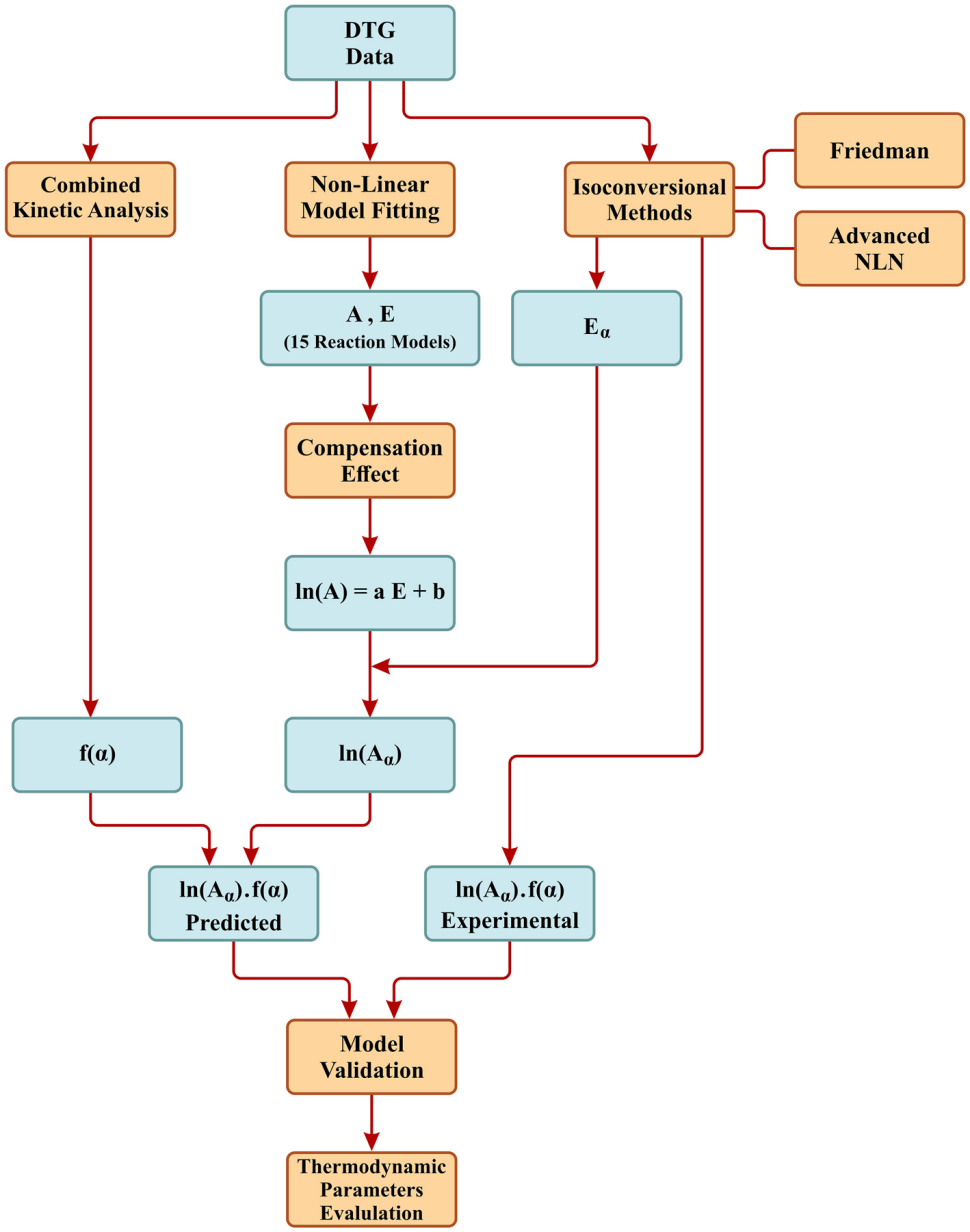
Procedural workflow of the kinetic analysis.

In this section, following a thorough examination and analysis of laboratory results, the investigation unfolds in a systematic sequence. Firstly, the outcomes related to the activation energy and the ‘*ln*(*f*(*α*)*A*_*α*_)’ quantity, derived through the application of the Friedman and ANIC isoconversional methods, are presented. Subsequently, the assessment of the reaction model is conducted using the CKA procedure. Employing the CE methodology, the values of the pre-exponential factor are scrutinized. Additionally, using both the CKA and CE results, the ‘*ln*(*f*(*α*)*A*_*α*_)’ quantity is computed and compared with the values obtained through the isoconversional methods. Finally, the thermodynamic parameters of the process are computed and examined using the final results in all three tested samples. The procedural workflow of the kinetic analysis in this study is shown in Fig. 1.

### Experimental and TG analysis

The summary of the experimental analysis of sugarcane residues is presented in Table 2 [In the table, the reported values represent the average of three replicate experiments, ‘ ± S.D.’ indicates standard deviation, ‘db.’ denotes the dry basis, fixed carbon calculated by difference (dry basis): 100-VM-Ash, and oxygen content calculated by difference (dry basis): 100-(C + H + N + Ash)]. These experimental results are derived from our recent study^1^.

**Table 2:** Experimental analysis of S.C.S., S.C.B., and S.B.P.

Quantity	S.C.S	S.C.B	S.B.P
Moisture (wt.%)	4.35 ± 0.55	4.68 ± 0.3	3.93 ± 0.29
Proximate analysis (wt. db.%)			
Volatile matter (V.M.)	77.50 ± 0.35	79.30 ± 0.36	77.30 ± 0.53
Fixed carbon (F.C.)	11.60 ± 0.65	10.95 ± 0.58	8.80 ± 1.42
Ash	10.90 ± 0.50	9.75 ± 0.69	13.90 ± 1.04
Ultimate analysis (wt. db.%)			
Carbon (C)	41.11 ± 0.07	43.56 ± 0.11	39.22 ± 0.06
Hydrogen (H)	5.61 ± 0.02	6.03 ± 0.02	5.36 ± 0.03
Nitrogen (N)	0.25 ± 0.00	0.20 ± 0.00	0.15 ± 0.00
Oxygen (O)	42.13 ± 0.05	40.46 ± 0.10	41.37 ± 0.05
Heating values (M.J./kg)			
HHV (db.)	16.86 ± 0.23	18.16 ± 0.15	16.20 ± 0.16
Chemical composition (wt. db.%)			
Holocellulose	65.08 ± 0.78	67.39 ± 0.30	63.29 ± 0.52
α-Cellulous	39.83 ± 0.81	39.42 ± 0.28	35.24 ± 0.20
Hemicellulose	25.25 ± 0.82	27.97 ± 0.28	28.05 ± 0.91
Acid insoluble lignin	23.57 ± 0.21	20.88 ± 0.09	19.09 ± 0.12
Extractive	3.71 ± 0.01	1.78 ± 0.12	3.99 ± 0.21

**Figure 2:**
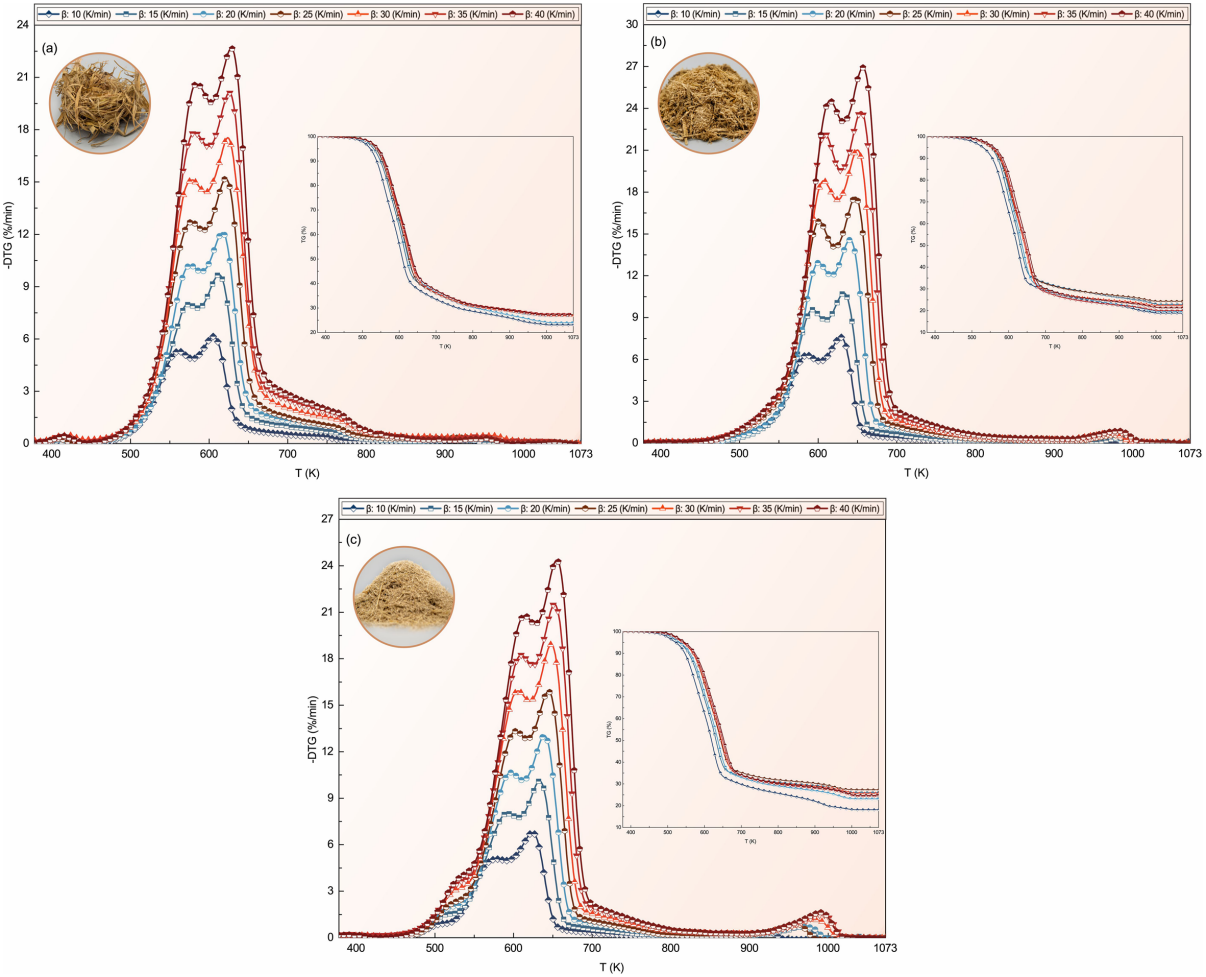
TG and DTG curves of (**a**) S.C.S., (**b**) S.C.B., and (**c**) S.B.P. at various heating rates.

The composition of biomass polysaccharides and their monosaccharide components significantly impact both the rate of thermal decomposition and the thermal stability of biomass^1^. Thermogravimetric analysis (TGA) findings (see Fig. 2) have allowed the categorization of the thermal decomposition of three biomass samples, namely S.C.S., S.C.B., and S.B.P., into four distinct stages^1^:

1. Dehydration Stage (< 423 K): This stage involves the removal of internal and external water from the biomass.
2. Torrefaction Stage (423–548 K): During this stage, extractives and a portion of hemicellulose undergo decomposition.
3. Active Pyrolysis Stage (548–673 K): In this zone, hemicellulose, cellulose, and a portion of lignin are decomposed. The derived thermogravimetry (DTG) curve typically exhibits two prominent peaks, with the dominant peak attributed to the devolatilization of cellulose.
4. Passive Pyrolysis Stage (673–1073 K): This stage primarily involves the decomposition of lignin.

According to Fig. 2 and Table 3 [In the table, ‘ ± S.D.’ indicates standard deviation], comparing weight changes at different temperature ranges, it was observed that S.C.S. and S.B.P. experienced greater weight loss than S.C.B. during the torrefaction stage. The weight loss in S.C.S. was mainly attributed to extractive materials, while in S.B.P., the type of hemicellulose and its interactions with other polysaccharides affected the rate of weight loss within this stage^1^. In the active pyrolysis zone, S.C.B. demonstrated the highest weight loss and thermal degradation rate due to its elevated levels of hemicellulose and cellulose content^1,41^.

**Table 3:** Temperature-dependent average weight loss of S.C.S., S.C.B., and S.B.P.

Material	Temperature zone (K)				Average total volatiles (wt.%)
	< 423	423–548	548–673	673–1073	
	Average weight loss %				
S.C.S	4.91 ± 0.59	7.55 ± 1.52	48.88 ± 1.62	12.18 ± 1.30	68.61 ± 1.17
S.C.B	4.89 ± 0.43	4.32 ± 1.42	58.02 ± 1.24	12.04 ± 1.49	74.38 ± 2.57
S.B.P	4.19 ± 0.32	6.27 ± 1.58	53.30 ± 0.69	11.48 ± 1.93	71.05 ± 2.69

The peak temperature analysis of the DTG profile for S.C.S., S.C.B., and S.B.P. at the different heating rates in the active pyrolysis zone is shown in Table 4. Accordingly, the decomposition rate and peak temperature are increased with increasing the heating rate for all samples. The results showed that the average first and second peak temperature (mainly related to the hemicellulose and cellulose, respectively) of S.C.B. is equal to 602 and 644 K and are higher than the average peak temperature of S.B.P. (i.e., 600 and 642 K) and S.C.S. (i.e., 576 and 619 K), respectively. Accordingly, a similar result can be obtained for the decomposition rate at the different heating rates as follows: S.C.S. < S.B.P. < S.C.B.

**Table 4:** DTG curve peak analysis of S.C.S., S.C.B., and S.B.P. at different heating rates.

Material	Initial sample weight (mg)	Heating rate (β) (K/min)	1st. Peak high (-%/min)	1st. Peak T (K)	2nd. Peak high (-%/min)	2nd. Peak T (K)
S.C.S	10.6803	10	5.29	561.76	6.14	605.18
	10.3332	15	7.97	572.70	9.69	613.09
	10.8083	20	10.21	575.41	12.09	616.92
	10.4703	25	12.70	577.03	15.19	621.09
	10.2390	30	15.11	579.70	17.54	623.43
	10.2092	35	17.81	581.46	20.13	625.40
	11.1882	40	20.61	584.49	22.67	628.03
S.C.B	11.4311	10	6.31	585.92	7.59	628.22
	10.3796	15	9.57	592.74	10.76	632.98
	10.4328	20	12.93	600.92	14.56	639.73
	10.7427	25	15.91	601.46	17.62	646.76
	10.9838	30	18.80	607.54	21.01	649.61
	10.8690	35	22.17	608.15	23.76	653.91
	10.9869	40	24.53	615.66	26.92	657.00
S.B.P	10.3505	10	5.07	578.62	6.77	624.31
	10.6431	15	8.03	591.90	10.11	633.24
	11.6852	20	10.66	596.55	13.05	639.34
	10.7675	25	13.34	603.36	15.90	644.75
	11.1169	30	15.91	606.58	18.91	648.68
	10.3912	35	18.24	609.69	21.53	651.75
	10.3616	40	20.77	614.29	24.35	654.34

### Isoconversional analysis

In this investigation, the kinetic parameters were determined employing the in-house kinetic calculation software known as XTKinetic, developed on the Python platform. The results of the linear regression parameters obtained using the Kissinger method are shown in Table 5 [In the table, the uncertainty ( ±) was determined using the traditional standard error approach with 95% confidence intervals^65^]. Additionally, Tables 6 and 7 [In the table, ${\Omega }_{\alpha }^{*}={\Omega }_{\alpha }-n\left(n-1\right)$] present outcomes for activation energies (*E*_*a*_) and *ln*(*f*(*α*)*A*_*α*_, along with their error metrics (i.e., R^2^ and ${\Omega }_{\alpha }$), for S.C.S., S.C.B., and S.B.P. samples determined using Friedman and ANIC methods. Both methodologies involved computing *E*_*a*_ within a conversion factor (*α*) range of 0.1–0.7 and a heating rate (*β*) of 10–40 K/min.

**Table 5:** Kissinger method results.

Material	*E*_*a*_ (kJ/mol)	ln (*A*) (1/s)	*R*^2^
S.C.S	176.473 ± 9.442	30.439 ± 1.834	0.999
S.C.B	146.911 ± 20.831	23.298 ± 3.889	0.985
S.B.P	138.749 ± 5.700	21.824 ± 1.068	0.999

**Table 6:** Friedman method results.

Material	S.C.S			S.C.B			S.B.P		
Conversion	*E*_*a*_ (kJ/mol)	*ln*(*f*(*α*)*A*_*α*_)	*R*^2^	*E*_*a*_ (kJ/mol)	*ln*(*f*(*α*)*A*_*α*_)	*R*^2^	*E*_*a*_ (kJ/mol)	*ln*(*f*(*α*)*A*_*α*_)	*R*^2^
0.10	170.87	31.222	0.977	144.195	23.926	0.991	135.542	22.531	0.998
0.15	179.14	32.656	0.958	151.320	25.239	0.994	127.493	20.566	0.996
0.20	180.21	32.522	0.954	155.869	26.009	0.991	130.988	21.098	0.997
0.25	180.14	32.125	0.965	161.754	26.993	0.988	139.624	22.650	0.997
0.30	182.77	32.249	0.971	169.122	28.197	0.987	149.388	24.365	0.991
0.35	188.78	33.030	0.978	177.694	29.557	0.986	158.249	25.818	0.982
0.40	194.45	33.709	0.985	184.176	30.436	0.985	164.036	26.592	0.974
0.45	196.26	33.630	0.992	185.207	30.235	0.984	163.859	26.177	0.974
0.50	194.11	32.822	0.996	181.123	29.093	0.980	160.747	25.242	0.978
0.55	191.29	31.939	0.996	175.159	27.671	0.977	156.855	24.236	0.979
0.60	190.59	31.494	0.995	172.339	26.904	0.976	155.066	23.683	0.976
0.65	192.14	31.448	0.991	174.540	27.090	0.975	156.522	23.747	0.963
0.70	198.97	32.296	0.978	184.964	28.755	0.972	163.591	24.799	0.931
Average	187.671	32.396	0.980	170.574	27.700	0.983	150.920	23.962	0.980

**Table 7:** Advanced NLN isoconverstional method results.

Material	S.C.S			S.C.B			S.B.P		
Conversion	*E*_*a*_ (kJ/mol)	*ln*(*f*(*α*)*A*_*α*_)	${\Omega }_{\alpha }^{*}$	*E*_*a*_ (kJ/mol)	*ln*(*f*(*α*)*A*_*α*_)	${\Omega }_{\alpha }^{*}$	*E*_*a*_ (kJ/mol)	*ln*(*f*(*α*)*A*_*α*_)	${\Omega }_{\alpha }^{*}$
0.10	169.580	31.013	0.204	143.320	23.668	0.084	137.260	22.932	0.011
0.15	179.680	32.853	0.368	151.400	25.234	0.051	127.160	20.488	0.034
0.20	179.680	32.479	0.437	154.430	25.710	0.071	131.200	21.142	0.023
0.25	179.680	32.089	0.295	161.500	26.942	0.100	138.270	22.409	0.020
0.30	182.710	32.309	0.254	169.580	28.296	0.113	147.360	24.037	0.077
0.35	188.770	33.113	0.194	176.650	29.367	0.130	157.460	25.794	0.162
0.40	194.830	33.867	0.133	182.710	30.183	0.145	163.520	26.652	0.227
0.45	195.840	33.616	0.078	184.730	30.205	0.145	164.530	26.470	0.249
0.50	193.820	32.836	0.036	180.690	29.095	0.152	160.490	25.331	0.191
0.55	191.800	32.114	0.033	175.640	27.865	0.160	156.450	24.282	0.163
0.60	189.780	31.423	0.033	172.610	27.063	0.186	156.450	24.071	0.164
0.65	191.800	31.499	0.063	172.610	26.823	0.190	156.450	23.887	0.271
0.70	197.860	32.281	0.174	183.720	28.611	0.225	162.510	24.822	0.532
Average	187.372	32.423	0.177	169.968	27.620	0.135	150.701	24.024	0.163

It is worth mentioning that, in this study, the conversion factors greater than 0.7 were not considered due to their potential non-linear behavior and the increased likelihood of precision loss, particularly in the vicinity of the TGA/DTG peak tail^20^. A critical observation suggests that an accurate assessment of the *E*_*a*_ dependency is achievable by approximating the temperature integral with a small Δ*α*, specifically 0.02^28,30^. Therefore, in our research, the computation was executed for every *α* value within the range of 0.1 to 0.7, utilizing a step size of 0.01.

**Figure 3:**
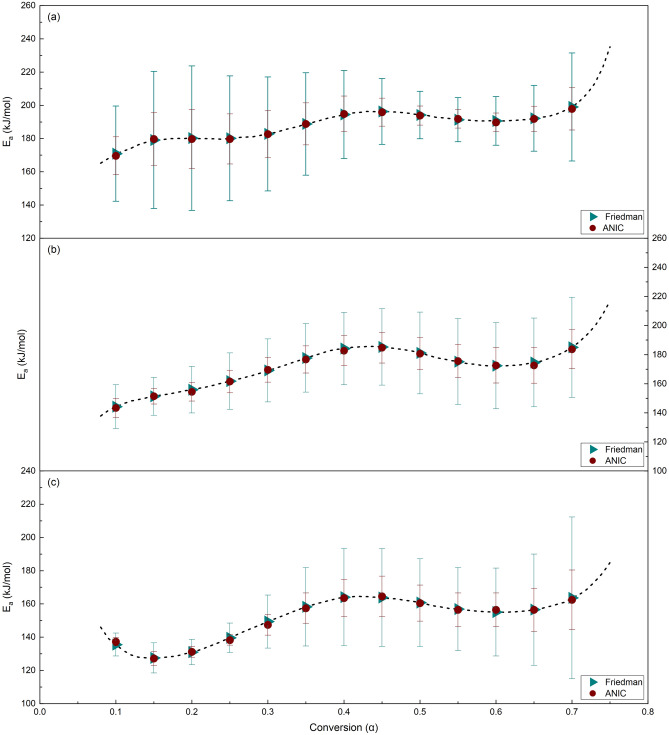
Dependence of activation energy (*E*_*a*_) on conversion factor (α) of (**a**) S.C.S., (**b**) S.C.B., and (**c**) S.B.P.

Furthermore, the uncertainty of the activation energy (*E*_*a*_) obtained from the Friedman method was estimated using the traditional linear regression standard error approach, in line with 95% confidence intervals^65^. This analysis was performed in conjunction with Vyazovkin and Sbirrazzuoli modifications^66^. Also, the uncertainty in the *E*_*a*_ value calculated by the ANIC method was assessed by applying the approach recommended by Vyazovkin and Wight^67^, incorporating 95% confidence intervals. Figure 3 depicts the dependency of the activation energy (*E*_*a*_) on the conversion factor and its uncertainty for three samples.

**Figure 4:**
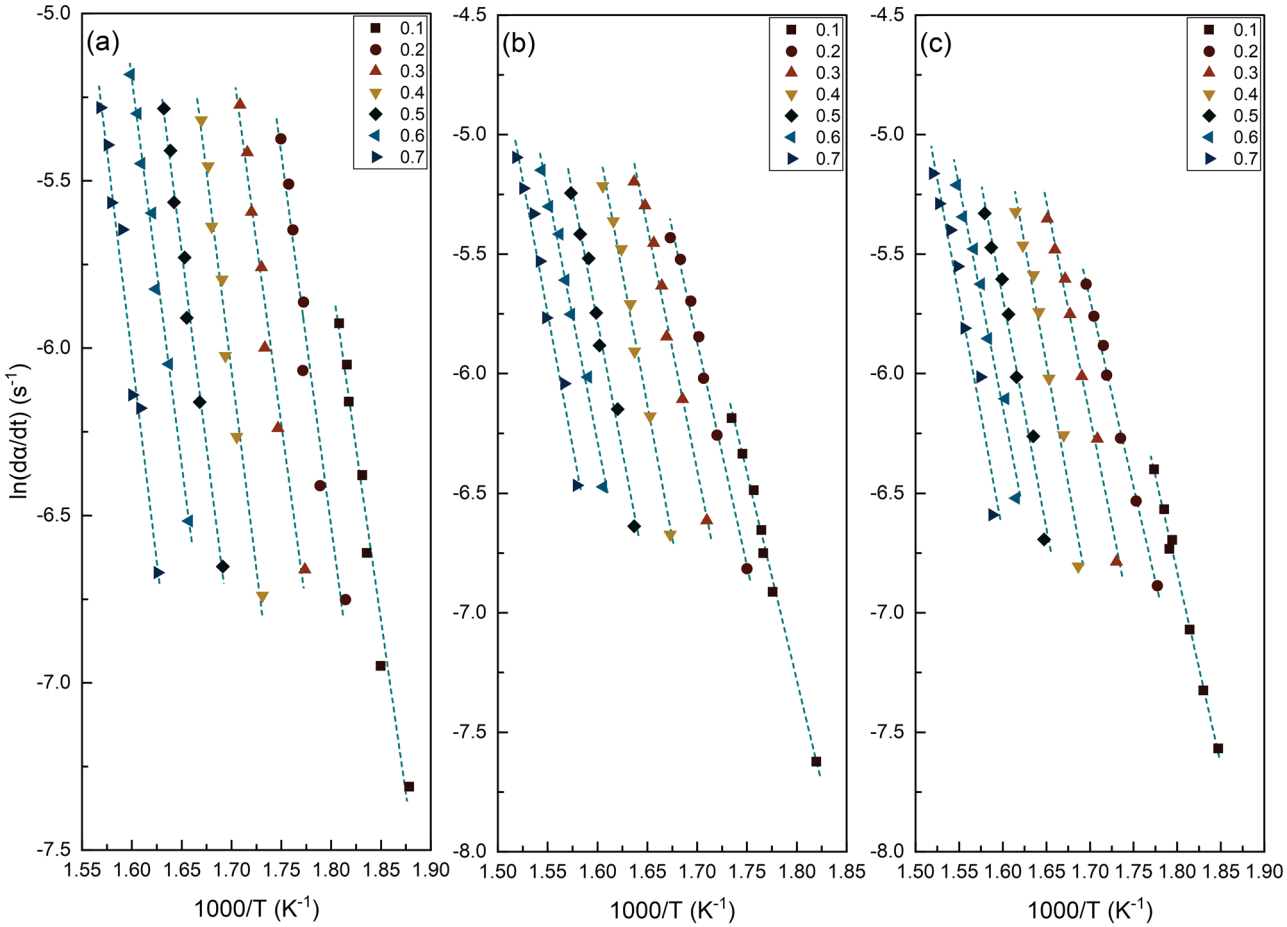
Friedman plot of (**a**) S.C.S., (**b**) S.C.B., and (**c**) S.B.P. at different conversion factor (*α*).

As can be observed in Fig. 3, the pyrolysis activation energies of each biomass sample increase until the conversion degrees of 0.45, then decrease slightly until the conversion degrees of 0.6 before increasing significantly for the conversion degrees higher than 0.7. The likelihood of an accelerated decomposition process for the primary composition, which approaches equilibrium at early stages, is inferred by the minor increase in activation energy (*E*_*a*_) for S.C.S. and S.C.B. before the conversion value of 0.2. It should be highlighted that this expedited degradation process may differ from the one at the start of the thermal conversion, which is mainly caused by the degradation of low-molecular composition^68^. The decrease in the activation energy (*E*_*a*_) for S.B.P. at *α* < 0.2 could be attributed to the type of hemicellulosic material present in this biomass^1,69,70^ as well as the depithing process^71^, which involves the lignin softening and rearrangement of fibers. Furthermore, the larger drop in activation energy (*E*_*a*_) for S.C.B. and S.B.P. at *α* > 0.45 may be attributed to the reduced lignin concentration of S.C.B. and S.B.P. compared to S.C.S. The limited deviation of activation energy (*E*_*a*_) values at 0.15 < *α* < 0.3 for S.C.S. indicates that the hemicellulose degradation mechanism in S.C.S. remains similar throughout the selected conversion range. Moreover, the deviation from linearity in the final conversions of S.C.S., S.C.B., and S.B.P. can be ascribed to intricate multi-step reaction mechanisms unfolding across a spectrum of temperatures under various heating rates, all influenced by heat and mass transport mechanisms^68,72^. The Friedman plot for S.C.S., S.C.B., and S.B.P. are shown in Fig. 4. The conversion factors of 0.1, 0.2, 0.3, 0.4, 0.5, 0.6, and 0.7 were employed in the regression analyses. All potential conversions produce almost parallel fitted lines, consistent with similar activation energy across the conversions. Besides, when the fitting lines are not parallel, it can be inferred that a shift from one set of reaction mechanisms to another has occurred^68,73^.

The findings of the Friedman method indicated that the activation energies of S.C.S., S.C.B., and S.B.P. are around 171–199 (avg., 188), 144–185 (avg., 170), and 136–164 (avg., 151) kJ/mol, respectively. As can be seen, the application of the ANIC method leads to results that are extremely close to the specified ranges. Specifically, Kissinger^74^ demonstrated that his approach causes the *E*_*a*_ values to be underestimated. The results presented in Table 5 confirm this proposition. Investigations show that the obtained *E*_*a*_ values are comparable and close to the values reported in the literature for thermal decomposition of S.C.S. and S.C.B.^41,60,72,73,75^. However, no analogous results were found for the pyrolysis of S.B.P. As shown in Fig. 3, if the conversion step (*Δα*) in the ANIC method is lower than 0.02 (here 0.01 is chosen), then the values of the activation energy and *ln*(*f*(*α*)*A*_*α*_) obtained will be quite near to those obtained from Friedman method, even though the trend and *E*_*a*_ values deviate significantly from Friedman method for larger values of *Δα* (e.g., *Δα* > 0.02).

### Kinetic model analysis

**Figure 5:**
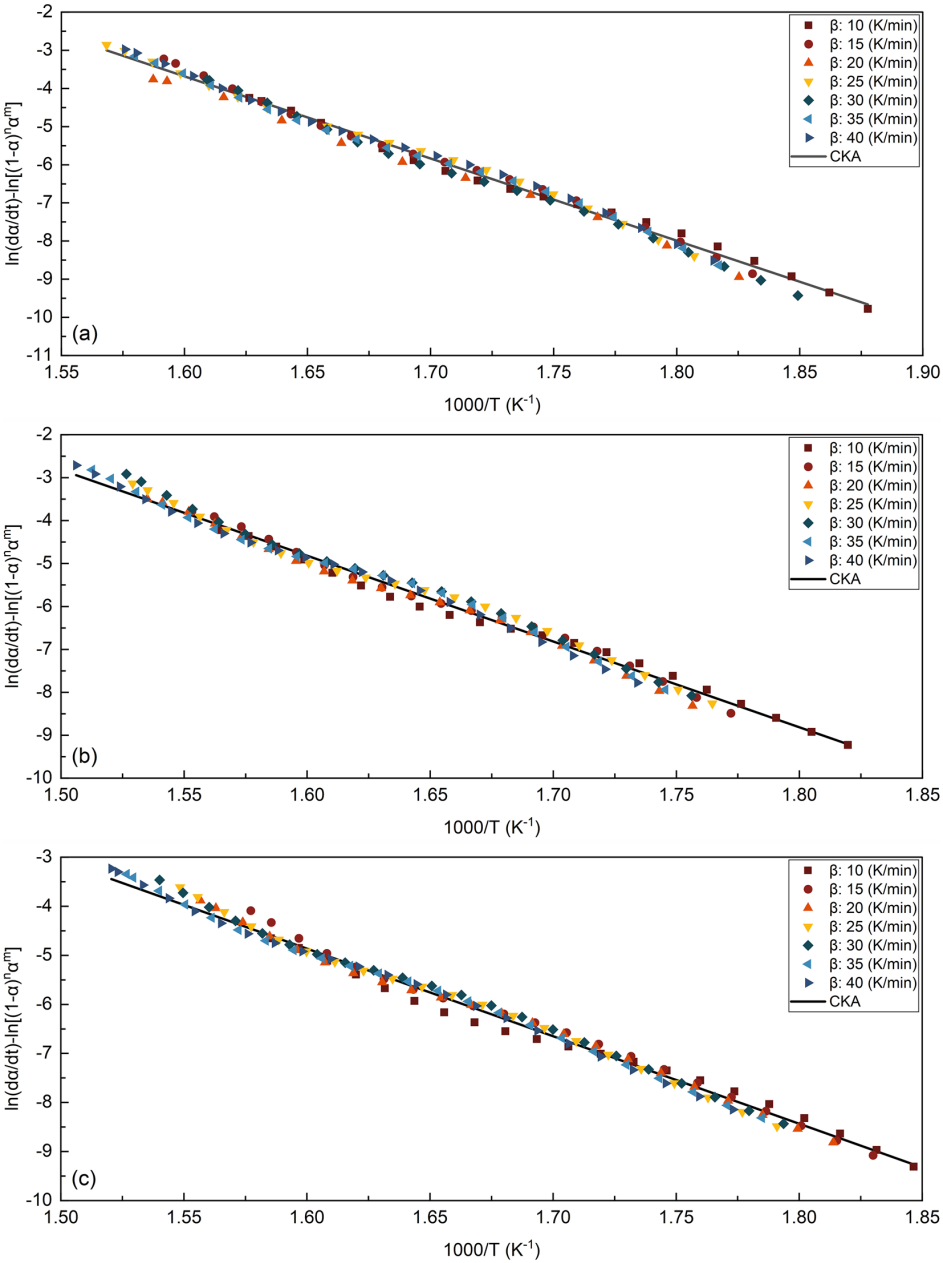
Combined kinetic analysis plot for (**a**) S.C.S., (**b**) S.C.B., and (**c**) S.B.P.

Table 8 [The uncertainty ( ±) was determined using the traditional standard error approach with 95% confidence intervals^65^] and Fig. 5 present the findings of the combined kinetic analysis (CKA) for S.C.S., S.C.B., and S.B.P. across seven heating rates. According to the results for S.C.B., a conversion factor (*α*) of 0.1–0.75 yields the highest R^2^ value, whereas, for S.C.S. and S.B.P., the corresponding ranges were 0.1–0.7. Comparing the results of activation energies in Table 6 and Table 8 show that the activation energies (*E*_*a*_) obtained using CKA for S.C.S., S.C.B., and S.B.P. differ by 4.78%, 2.62%, and 1.66%, from the average Friedman method results, respectively. Similarly, comparable outcomes can be achieved through the application of the ANIC method. In this context, it becomes evident that the outcomes from both the Friedman and ANIC methods are in close agreement. Based on this and the findings already described, also by ignoring the calculation of *c* in Eq. (22), the optimization outcomes will be analysed and compared in the following sections.

**Table 8:** Combined kinetic analysis and the modified truncated Sestak-Berggren kinetic equation optimization parameters.

Material	*E*_*a*_ (kJ/mol)	*ln*(*cA*) (1/s)	*n*	*m*	*R*^*2*^
S.C.S	179.104 ± 1.165	30.787 ± 0.466	2.36821 ± 0.06572	− 1.19136 ± 0.03989	0.944
S.C.B	166.213 ± 2.232	27.172 ± 0.434	1.93872 ± 0.05229	− 0.78519 ± 0.03617	0.940
S.B.P	148.453 ± 1.749	23.706 ± 0.343	1.86746 ± 0.05431	− 0.84760 ± 0.03358	0.950

### Compensation effect analysis

In order to determine the pre-exponential factor, TGA data and a model-fitting method were used to evaluate 15 reaction models presented in Table 1 in accordance with the compensation effect (CE) study described in Section "Determining the pre-exponential factor". The statistical parameters mentioned in Table 9 [In the table, in Eqs. (28)–(34), *n*: number of data points, ${\omega }_{i}$: the weight corresponding to ith value of the variable, *p*: number of model parameters, ${y}_{i}$: ith value in a sample, ${\widehat{y}}_{i}$: ith value of the variable to be predicted, $\overline{y }$: mean value of a sample] were utilized to compare the obtained results. It was also shown that one of the most useful metrics for the model selection and statistical analysis is the normalized root means square error (nRMSE) parameter. Accordingly, besides Bayesian information criterion (BIC) and Akaike's information criterion (AIC), nRMSE has also been utilized as an accuracy metric in the model selection^76–78^. In this analysis, the model accuracy has been marked as “outstanding” when nRMSE was less than 10%, “good” when nRMSE was between 10 and 20%, “fair” when nRMSE was between 20 and 30%, and “poor” when nRMSE was greater than 30%^77^.

**Table 9:** Statistical metrics for evaluation of the model accuracy.

Abbreviations	Description	Formula	Eq	Ref
*AIC*	Akaike’s information criterion	$n+n{\text{ln}}\left(2\pi \right)+n ln\left(\frac{RSS}{n}\right)-\sum_{i=1}^{n}ln{\omega }_{i}+2(p+1)$	(28)	^79,80^
*BIC*	Bayesian information criterion	$n+n\mathit{ln}\left(2\pi \right)+n ln\left(\frac{RSS}{n}\right)-\sum_{i=1}^{n}ln{\omega }_{i}+ln(n)(p+1)$	(29)	^79,80^
*RSS*	Residual sum of squares	$\sum_{i=1}^{n}{\left({y}_{i}{-\widehat{y}}_{i}\right)}^{2}$	(30)	^79,81^
*TSS*	Total sum of squares	$\sum_{i=1}^{n}{\left({y}_{i}-\overline{y }\right)}^{2}$	(31)	^79,81^
*nRMSE*	Normalized root means square error	$\frac{1}{\left|\overline{y }\right|}.\sqrt{\sum_{i=1}^{n}\frac{{\left({y}_{i}-{\widehat{y}}_{i}\right)}^{2}}{n}}$	(32)	^82^
*R*^2^	Coefficient of determination	$1-\frac{RSS}{TSS}=1-\frac{\sum_{i=1}^{n}{\left({y}_{i}-{\widehat{y}}_{i}\right)}^{2}}{\sum_{i=1}^{n}{\left({y}_{i}-\overline{y }\right)}^{2}}$	(33)	^81,83^
*RE*	Relative error	$\left|\frac{{y}_{i}-{\widehat{y}}_{i}}{{y}_{i}}\right|$	(34)	-

To achieve the maximum possible R^2^ from the model-fitting procedure, the optimal range of conversion factor (*α*) of TGA data for S.C.S. was 0.1–0.7, while the corresponding ranges for S.C.B. and S.B.P. were 0.1–0.75 and 0.1–0.8, respectively.

In the present study, the best set of the calculated model-fitting kinetic parameters based on the heating rate that leads to the appropriate CE-dependent parameters was chosen. This decision was made based on comparing the relative error between *ln*(*f*(*α*)*A*_*α*_) calculated from CE parameters at different heating rates and the reaction model obtained from CKA with the results obtained from the Friedman and ANIC method. On the basis of this information, the heating rate of 15 K/min was found to produce the best results considering the agreement with the TGA experiments. It should be noted that, a comparative analysis revealed that the values of the pre-exponential factor (*ln*(*A*_*α*_)), obtained through the compensation effect methodology and involving fitting 15 reaction models at different heating rates, exhibit a standard deviation ranging from 0.02 to 0.1 for all three samples.

**Figure 6:**
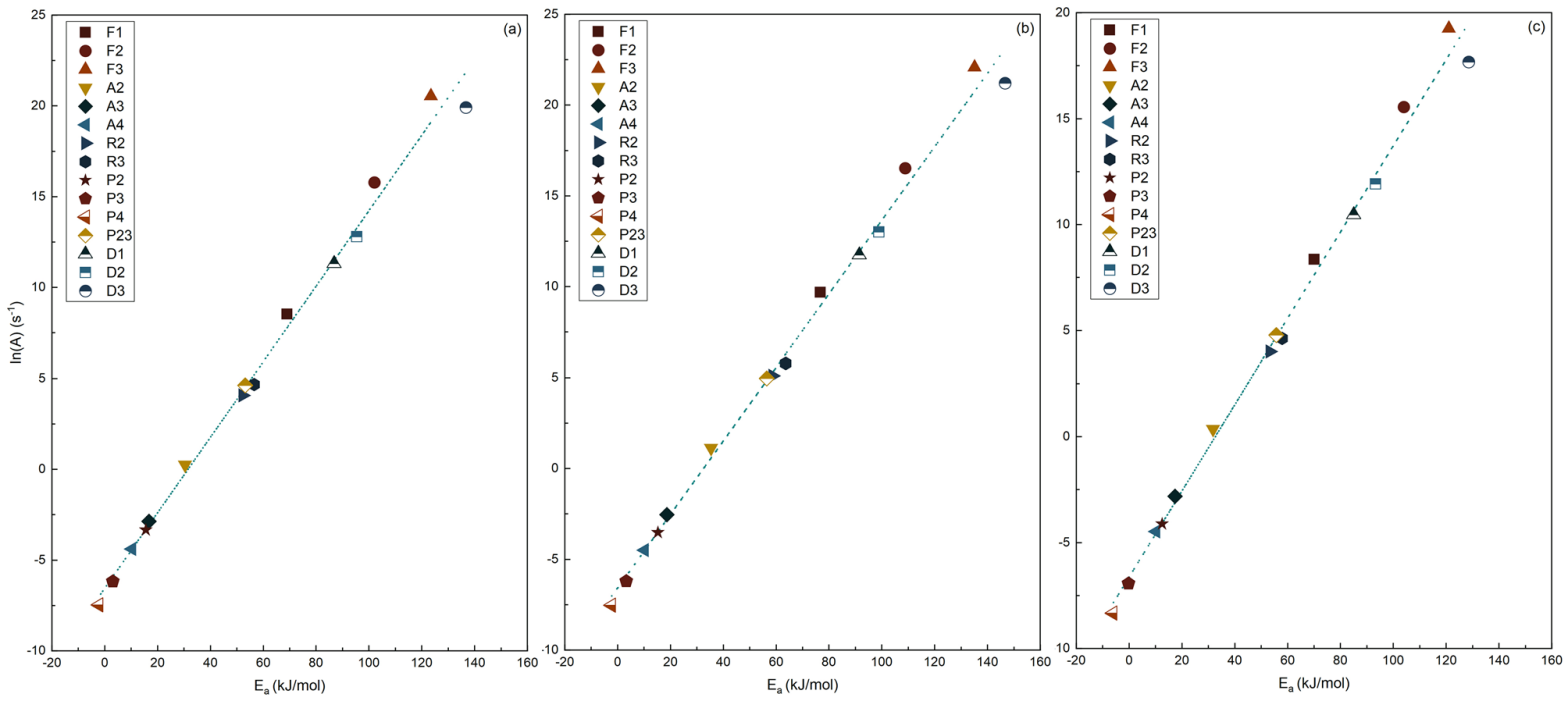
Compensation effect plot (dot line) for (**a**) S.C.S., (**b**) S.C.B., and (**c**) S.B.P. The data points on the graph represent the *ln*(*A*) and *E*_*a*_ values that were determined for the 15-reaction models in Table 1 using a heating rate of 15 K/min.

Figure 6 and Table 10 illustrate the CE plot and the outcomes of the model-fitting method for the heating rate of 15 K/min across all three samples. Also, Table 11 provides the acquired CE parameters. From Table 10 and in accordance with the defined range for nRMSE, it can be seen that the results of the reaction models F1, F2, A2, A3, and A4 for S.C.S., F1, F2, A2, A3, A4, R2, and R3 for S.C.B., and F1, A2, A3, A4, R2, R3 and D3 for S.B.P., have “good” accuracy compared to other models, while the results from other models do not. It is worth noting that the related conclusions can be deduced for other heating rates.

**Table 10:** Non-linear model-fitting results for S.C.S., S.C.B., and S.B.P. with 15-reaction models based on a heating rate of 15 K/min.

Material	Reaction model	*ln*(*A*_*α*_)	*E*_*α*_	*R*^2^	*nRMSE%*	*AIC-AIC*_*min*_	*BIC-BIC*_*min*_
S.C.S	F1	9.681	76.732	0.9709	17.6730	8.5	8.5
	F2	16.520	108.900	0.9718	17.4052	0.0	0.0
	F3	22.080	135.100	0.9541	21.6672	135.1	135.1
	A2	1.120	35.400	0.9684	18.4071	31.1	31.1
	A3	− 2.541	18.697	0.9705	17.7806	11.9	11.9
	A4	− 4.503	10.200	0.9668	18.8681	44.9	44.9
	R2	5.110	58.700	0.9495	23.2733	161.5	161.5
	R3	5.780	63.600	0.9592	20.9215	102.3	102.3
	P2	− 3.527	15.240	0.8970	33.2317	359.6	359.6
	P3	− 6.203	3.267	0.9009	32.5984	348.9	348.9
	P4	− 7.532	− 2.297	0.9036	32.1589	341.4	341.3
	P23	4.960	56.400	0.8769	36.3349	409.2	409.2
	D1	11.750	91.500	0.8936	33.7771	368.6	368.6
	D2	13.010	98.900	0.9163	29.9691	302.1	302.1
	D3	21.210	146.700	0.9564	21.6264	120.7	120.7
S.C.B	F1	8.520	69.001	0.9827	14.3186	0.0	0.0
	F2	15.786	102.059	0.9741	17.5437	121.9	121.8
	F3	20.550	123.500	0.9618	21.2895	238.0	237.9
	A2	0.251	30.450	0.9818	14.7059	16.1	16.0
	A3	− 2.863	16.707	0.9825	14.3949	3.2	3.1
	A4	− 4.390	10.301	0.9800	15.4029	43.8	43.7
	R2	4.072	52.225	0.9683	19.4061	182.5	182.4
	R3	4.670	56.600	0.9752	17.1530	108.4	108.3
	P2	− 3.343	15.578	0.9324	28.3292	409.4	409.4
	P3	− 6.184	3.129	0.9433	25.9326	356.4	356.3
	P4	− 7.479	− 2.178	0.9476	24.9316	332.8	332.7
	P23	4.620	53.200	0.9112	32.4714	491.3	491.2
	D1	11.320	86.805	0.9298	28.8744	420.9	420.8
	D2	12.790	95.400	0.9511	24.0983	312.4	312.3
	D3	19.910	136.700	0.9648	20.4260	213.2	213.1
S.B.P	F1	8.349	70.109	0.9790	13.7168	0.0	0.0
	F2	15.550	104.000	0.9503	21.0929	259.9	259.9
	F3	19.260	121.000	0.9163	27.3743	417.4	417.4
	A2	0.361	31.820	0.9742	15.2008	62.1	62.1
	A3	− 2.819	17.365	0.9731	15.5316	75.1	75.1
	A4	− 4.473	10.182	0.9700	16.3782	107.1	107.1
	R2	4.008	53.260	0.9744	15.1507	60.1	60.1
	R3	4.630	57.900	0.9789	13.7309	0.6	0.7
	P2	− 4.122	12.455	0.9301	25.0066	362.7	362.8
	P3	− 6.937	− 0.189	0.9449	22.2102	291.1	291.1
	P4	− 8.335	− 6.093	0.9498	21.1969	262.9	262.9
	P23	4.780	55.700	0.9185	27.0102	409.3	409.3
	D1	10.468	84.994	0.9179	27.1075	411.4	411.5
	D2	11.920	93.300	0.9517	20.7964	251.4	251.4
	D3	17.669	128.501	0.9784	13.8907	7.6	7.6

**Table 11:** Compensation effect parameters derived from 15- and 4-reaction models.

Number of models	Material	*a*	*b*	*R*^*2*^
15-Reaction models	S.C.S	0.207797	− 6.552594	0.993
	S.C.B	0.202302	− 6.573615	0.994
	S.B.P	0.203590	− 6.636447	0.993
4-Reaction models	S.C.S	0.218964	− 6.543099	0.999
	S.C.B	0.212267	− 6.544713	0.999
	S.B.P	0.213075	− 6.542448	0.999

The AIC, BIC, and R^2^ all confirm the accuracy evaluation. The results indicate that nRMSE can be used to select the optimal model for all three samples, whereas the level of empirical support of the model (e.g., AIC-AIC_min_) with different selection ranges, as well as the R^2^, could only verify the selected model. Additionally, while certain models assessed in this work have an AIC-AIC_min_ that does not meet the threshold of acceptability (less than 10), it has been shown that for the non-nested models, this threshold might be greater^79^. It should be emphasized that although the R^2^ comparison results are compatible with other statistical metrics, caution should still be taken when relying on R^2^ alone since it is defined as a pseudo-R^2^^83^ and is not directly attainable from the nonlinear optimization techniques.

### Kinetic results validation

A comparison of the results obtained in the previous three sections reveals that the *ln*(*f*(*α*)*A*_*α*_) values, calculated using the CE and CKA procedures for S.C.S., S.C.B., and S.B.P. are, on average, approximately 5% higher, 6% higher, and 7% lower than those obtained through Friedman and ANIC methods, respectively. However, the trend of *ln*(*f*(*α*)*A*_*α*_) calculated based on *ln*(*A*_*α*_) derived from the chosen reaction models and that of *f*(*α*) produced by CKA are consistent with the outcomes obtained using these methods. Furthermore, according to Sbirrazzuoli^31^, using the 4-reaction models Avrami-Erofeev (A2, A3, A4) and Mampel (F1) for CE parameter computation can result in trustworthy values. This study demonstrates that while all of the 4-reaction models are among all of the selected reaction models, calculating *ln*(*f*(*α*)*A*_*α*_) with the 4-reaction approach could increase its value by as much as 18% for S.C.S., 6% for S.C.B., and 20% for S.B.P. compared to the *ln*(*f*(*α*)*A*_*α*_) obtained using Friedman and ANIC methods.

In general, it can be stated that using a 4-reaction or the selected reaction model based on the statistical metrics of the model-fitting findings to calculate the CE parameters yields the final results of *ln*(*f*(*α*)*A*_*α*_) with the same trend as those of the model-free methods. However, the final values of *ln*(*f*(*α*)*A*_*α*_) might be produced with an absolute relative error of 5–20% due to the type of raw material, the accuracy of the experimental results, and the error-prone nature of the non-linear optimization approaches. This could be supported by similar findings in the published literature^84^.

According to the provided explanations and the experimental TG data of the present study (see Fig. 2), the optimal *ln*(*f*(*α*)*A*_*α*_) is resulted by selecting all 15 reaction models for CE parameters computation and employing the CKA reaction model for *f*(*α*) calculation.

**Figure 7:**
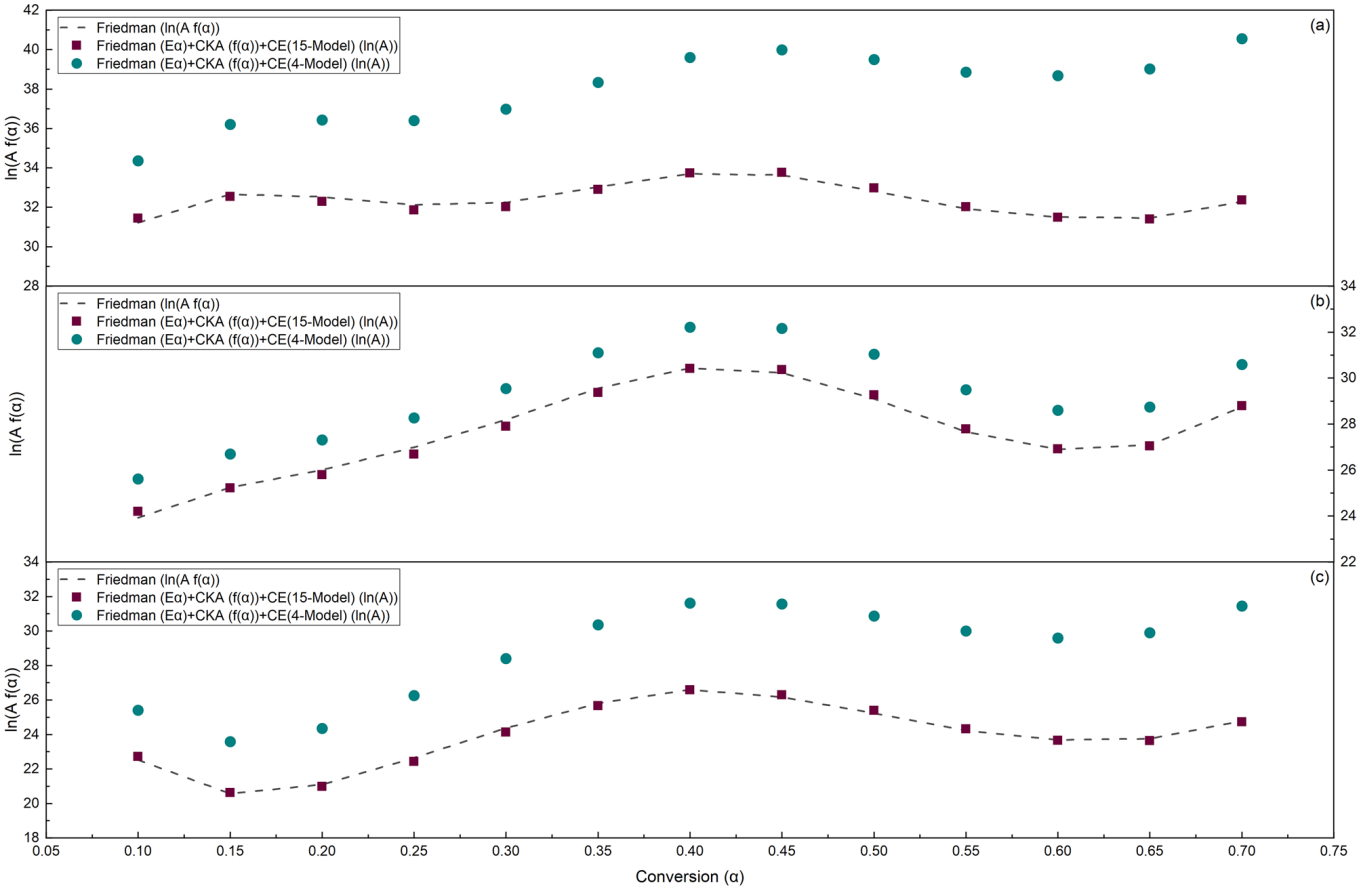
Comparison of *ln*(*f*(*α*)*A*_*α*_) based on the Friedman method and CE + CKA results for (**a**) S.C.S., (**b**) S.C.B., and (**c**) S.B.P.

Assuming that *c* is equal to one, Fig. 7 and Table 12 depict the comparison between the results obtained from the Friedman method (see Table 6) and the computed value for the quantity *ln*(*f*(*α*)*A*_*α*_) with CE-CKA findings. To calculate *ln*(*f*(*α*)*A*_*α*_), *A*_*α*_ is computed using Eq. (20), with the CE parameters obtained from the 15 and 4-reaction models, as shown in Table 11. Additionally, values of *f*(*α*) are determined based on the CKA results presented in Table 8 and Eq. (21).

**Table 12:** Comparison of *ln*(*f*(*α*)*A*_*α*_) values obtained from CE (15-reaction models)—CKA procedure and the Friedman method results.

Material	S.C.S				S.C.B				S.B.P			
Conversion	*f*(*α*)	*ln*(*A*_*α*_)	*ln*(*f*(*α*)*A*_*α*_)	*RE%*	*f*(*α*)	*ln*(*A*_*α*_)	*ln*(*f*(*α*)*A*_*α*_)	*RE%*	*f*(*α*)	*ln*(*A*_*α*_)	*ln*(*f*(*α*)*A*_*α*_)	*RE%*
0.10	12.106	28.954	31.448	0.723	4.971	22.597	24.201	1.151	5.783	20.959	22.714	0.812
0.15	6.523	30.673	32.548	0.330	3.237	24.039	25.213	0.103	3.686	19.320	20.624	0.285
0.20	4.011	30.895	32.284	0.732	2.296	24.959	25.790	0.841	2.579	20.031	20.979	0.562
0.25	2.639	30.881	31.851	0.851	1.700	26.149	26.680	1.159	1.892	21.790	22.427	0.984
0.30	1.803	31.425	32.015	0.726	1.289	27.640	27.894	1.076	1.425	23.777	24.132	0.955
0.35	1.259	32.675	32.906	0.376	0.989	29.374	29.363	0.653	1.089	25.581	25.667	0.584
0.40	0.889	33.853	33.735	0.076	0.763	30.686	30.415	0.071	0.838	26.760	26.582	0.036
0.45	0.628	34.229	33.765	0.401	0.587	30.894	30.362	0.419	0.644	26.724	26.284	0.407
0.50	0.442	33.783	32.967	0.443	0.450	30.068	29.268	0.604	0.493	26.090	25.383	0.560
0.55	0.308	33.196	32.017	0.246	0.340	28.861	27.783	0.403	0.374	25.298	24.313	0.319
0.60	0.210	33.051	31.490	0.013	0.253	28.291	26.915	0.044	0.279	24.933	23.655	0.118
0.65	0.139	33.373	31.400	0.152	0.183	28.736	27.039	0.187	0.203	25.230	23.634	0.472
0.70	0.088	34.793	32.366	0.219	0.128	30.845	28.791	0.125	0.143	26.669	24.723	0.306
Average	2.388	32.445	32.369	0.407	1.322	27.934	27.670	0.526	1.494	24.089	23.932	0.492

Based on these findings, it can be concluded that the average relative error for *ln*(*f*(*α*)*A*_*α*_) estimated using the CKA and CE methods for S.C.S., S.C.B., and S.B.P., compared to the values obtained using the Friedman method, is 0.407%, 0.526%, and 0.492%, respectively. The application of ANIC method yields comparable results; however, there is a slight increase in the average relative error for the estimated *ln*(*f*(*α*)*A*_*α*_), which is 0.507%, 0.565%, and 0.698% for S.C.S., S.C.B., and S.B.P., respectively.

### Thermodynamic parameter evaluations

To calculate the thermodynamic parameters, it is necessary to determine the reaction's kinetic characteristics at first and then assess the reaction's maximum peak or the decomposition temperature. The maximum peak temperature (T_p_) value can be obtained in a few different ways: (1) using the highest possible degradation temperature in the DTG curve at each heating rate or at the lowest heating rate, (2) by taking the average of the highest possible temperatures across all heating rates^85,86^. In this research, the maximum peak temperature is considered corresponding to β → 0, and it is determined by solving the quadratic equation, a_0_ + a_1_.β + a_2_.β^2^, where a_0_, a_1,_ and a_2_ are the numerical coefficients, and the reciprocal value of the a_0_ coefficient is identical to T_p_ at β → 0^68,87,88^. In this regard, the value of maximum peak temperature according to Table 4 for S.C.S., S.C.B., and S.B.P. has been calculated as 591.38, 612.13, and 605.52 K, respectively.

Table 13 presents the average values of ∆S^*^, ∆H^*^, and ∆G^*^ that were obtained by two distinct methods (Method I and II) based on the findings of the Friedman method. It should be noted that similar outcomes to those of the Friedman method could be obtained using the ANIC method.

**Table 13:** Average thermodynamic parameters based on the Friedman method.

Material	S.C.S			S.C.B			S.B.P		
Conversion	*ΔS* (J/mol.K)	*ΔH* (kJ/mol)	*ΔG* (kJ/mol)	*ΔS* (J/mol.K)	*ΔH* (kJ/mol)	*ΔG* (kJ/mol)	*ΔS* (J/mol.K)	*ΔH* (kJ/mol)	*ΔG* (kJ/mol)
0.10	− 14.039	165.954	174.257	− 67.178	139.105	180.227	− 80.454	130.508	179.224
0.15	0.254	174.227	174.077	− 55.193	146.231	180.016	− 94.101	122.459	179.439
0.20	2.102	175.297	174.054	− 47.541	150.780	179.881	− 88.175	125.954	179.346
0.25	1.983	175.228	174.055	− 37.643	156.664	179.707	− 73.532	134.590	179.115
0.30	6.510	177.848	173.998	− 25.250	164.032	179.488	− 56.978	144.353	178.854
0.35	16.901	183.862	173.867	− 10.830	172.605	179.234	− 41.954	153.214	178.618
0.40	26.696	189.531	173.744	0.071	179.086	179.043	− 32.141	159.001	178.463
0.45	29.822	191.341	173.705	1.806	180.118	179.012	− 32.442	158.824	178.468
0.50	26.114	189.195	173.751	− 5.064	176.033	179.133	− 37.718	155.712	178.551
0.55	21.232	186.369	173.813	− 15.095	170.070	179.310	− 44.317	151.821	178.655
0.60	20.027	185.671	173.828	− 19.839	167.249	179.393	− 47.350	150.032	178.703
0.65	22.705	187.222	173.794	− 16.136	169.451	179.328	− 44.882	151.487	178.664
0.70	34.507	194.053	173.646	1.397	179.874	179.019	− 32.896	158.556	178.475
Average	14.986	182.754	173.891	− 22.807	165.484	179.445	− 54.380	145.885	178.814

The values of ∆S^*^ and ∆H^*^ are seen to increase up to the range of conversion 0.45, then decrease relatively up to the conversion reaches of 0.6, after which they rise again. In contrast, ∆G^*^ exhibits a relative decline up to the conversion range of 0.45, then increases up to the range of 0.6 before declining again. Singh et al.^89^ have also observed a similar trend in this regard.

The value of ∆S^*^ signifies the extent to which a reaction tends to be in either the transition state or the ground state. A decreased or negative value of ∆S^*^ indicates that the reaction can proceed with less energy and difficulty. In such cases, ∆S^*^ often suggests an associative mechanism in which two reactants form a single active complex^90^.

∆S^*^ values close to zero (e.g., S.C.B.) during pyrolysis indicate that the feedstock experienced only slight chemical or physical change, resulting in a new condition close to its thermodynamic equilibrium. A low value of ∆S^*^ lengthens the time required for the pyrolysis reaction (referred to as "slow" reactions)^91,92^.

Larger negative values of ∆S^*^ (e.g., S.B.P.) indicate that the degree of disorder in the products is significantly lower compared to the initial reaction state, while positive values reveal the opposite^93^. In these circumstances, the reaction can involve a bimolecular step and is most likely a second-order reaction. ∆S^*^ in such instances indicates the entropy loss caused by the unification of the two reaction partners into a single transition state^90^. The devolatilization stage of all three samples produced negative ∆S^*^ values, which is a significant finding. A high or positive value of ∆S^*^ indicates that the activated complex, which is about to dissociate, has a high reactivity (associated with “fast” reactions) and is distant from the thermodynamic equilibrium state. Naturally, if the value of ∆S^*^ is more positive (e.g., S.C.S.), a fast reaction can have a higher activation energy^90–92^.

A significant factor in assessing whether a decomposition process is endothermic or exothermic is the value of the enthalpy change parameter ∆H^*^, which may be positive or negative. Moreover, ∆H^*^ is an important thermodynamic parameter for determining the energy required to convert the biomass into bioenergy products. Positive values of ∆H^*^ indicate that an external heat source is necessary for biomass pyrolysis to produce biofuels and bio-based compounds, as is typical of an endothermic reaction^92^. The activation energies are consistent with the value of ∆H^*^ being equal to the difference between the reagent and the activated complex^94^. The difference between activation energy (*E*_*a*_) values and ∆H^*^ reveals the likelihood of the pyrolysis reaction^93^. More specifically, a lower ∆H^*^ value suggests that the product formation was easier, whereas a larger ∆H^*^ value shows that the product formation was more complex^95^.

∆G^*^ is a parameter that reveals the amount of energy available from the feedstock throughout the pyrolysis process. It is also used to determine the spontaneity of the decomposition process, which is another essential factor^92–95^. Positive results for ∆G^*^ and ∆H^*^ in the thermodynamic parameters of S.C.S., S.C.B., and S.B.P. support the conclusion that the pyrolysis process is non-spontaneous, endothermic, and the event was also endergonic.

## Conclusions

This research explored the thermo-kinetic characteristics of sugarcane bagasse pith (S.B.P.) and conducted a comparative evaluation with two other significant sugarcane residues, namely sugarcane straw (S.C.S.) and sugarcane bagasse (S.C.B.), throughout the pyrolysis process. The study utilized rigorous thermogravimetric analysis and kinetic computations. Key findings include the robust agreement between the ANIC and Friedman methods, demonstrating consistency in *ln*(*f*(*α*)*A*_*α*_) and activation energy values. Furthermore, the study demonstrates that if a small step size (e.g., Δ*α* ≈ 0.01) is chosen for the ANIC method, the activation energy range calculated for S.C.S. (171–199 kJ/mol), S.C.B. (144–185 kJ/mol), and S.B.P. (136–164 kJ/mol) using Friedman's method were vin close agreement with ANIC results.

The method of calculating the *ln*(*f*(*α*)*A*_*α*_) quantity using two different methodologies, including CE and CKA, and comparing the results with those of the isoconversional method, along with accuracy assessments, exhibits scalability and precision in our approach. Additionally, the study shows that the application of different reaction models in the CE method to obtain the pre-exponential factor may lead to final results of *ln*(*f*(*α*)*A*_*α*_) with an absolute relative error of 5–20%.

Thermogravimetric analysis in the study reveals an accelerated decomposition process in the early stages, shaped by factors such as low-molecular composition and the influence of hemicellulosic material, providing qualitative insights into the distinctive thermal characteristics of each sample.

Thermodynamic analysis affirms the non-spontaneous and endothermic nature of pyrolysis for S.B.P., S.C.S., and S.C.B. Moreover, a distinct trend is revealed through the results obtained from activation energy and thermodynamic calculations, indicating that S.B.P. has lower thermal stability compared to S.C.B. and S.C.S. These findings align with physicochemical characterizations, emphasizing operational considerations for samples and highlighting the bioenergy potential of S.B.P., S.C.S., and S.C.B.

## Data Availability

It should be justified that “All data generated or analyzed during this study are included in this published article”.
